# Mothers and fathers of pre-school children: a study on parenting stress and child’s emotional-behavioral difficulties

**DOI:** 10.1007/s12144-022-03599-6

**Published:** 2022-08-23

**Authors:** Carmen Trumello, Giulia Ballarotto, Piera Ricciardi, Marinella Paciello, Valentina Marino, Mara Morelli, Renata Tambelli, Alessandra Babore

**Affiliations:** 1 Department of Psychological, Health and Territorial Sciences, University G. d’Annunzio of Chieti, Chieti, Italy; 2 Department of Dynamic and Clinical Psychology, and Health Studies, Sapienza University of Rome, via degli Apuli 1, 00185 Rome, Italy; 3 Faculty of Psychology, International Telematic University Uninettuno, Rome, Italy

**Keywords:** Parenting stress, Fathers, Pre-school children, Psychopathological risk

## Abstract

The present study aimed to analyze the differences between maternal and paternal parenting stress and children’s behavioral functioning, as determined by teacher and parent reports. In addition, it sought to evaluate the presence of clusters based on parenting stress and to determine whether perceptions of children’s behavioral functioning varied across these clusters. The sample was composed of the parents and teachers of N = 201 children. Parents completed a self-report questionnaire on parenting stress and parents and teachers filled out report-form questionnaires assessing children’s emotional and behavioral functioning. The results showed that mothers had higher levels of parenting stress than fathers, and both parents reported more prosocial behavior in children than did teachers. Furthermore, middle stressed parents had children who expressed more prosocial behavior than did parents in other clusters. The findings also showed that parenting stress influenced partners’ perceptions of children’s behavioral functioning. The multiplicity of child observers facilitated a better understanding of how parenting stress, linked to parent–child interactions, may play an important role in shaping parents’ perceptions of their children.

Research suggests that parenting stress has a significant impact on children’s development, both directly and indirectly (Crnic & Ross, 2017; Ward & Lee, 2020). For instance, several studies have found that high levels of parenting stress can negatively influence parents’ interactions with their children (Babore et al., 2014; Costa et al., 2006; Porreca et al., 2018; Trumello et al., 2021; Wright & Edginton, 2016). Parent–child interactions form the foundation for children’s positive social behaviors development, which are important for many domains of functioning (Gryczkowski et al., 2010; Pallini et al., 2018). In this vein, while several studies have focused on the influence of maternal parenting stress in the perinatal period, few studies have investigated the role of paternal parenting stress during early childhood (Cimino et al., 2018; Webb et al., 2018; Wilson & Prior 2011). The present study aimed to separately investigate the influence of maternal and paternal parenting stress on children’s behavioral functioning.

## Maternal and paternal parenting stress

Deater-Deckard (1998) defined parenting stress as “the stress reaction to the demands of being a parent” (p. 314). Additionally, given that parenting requires effort from both parents, parenting stress may also result from the interaction between maternal and paternal parenting stress (Castel et al., 2016; McBride et al., 2002). Some studies have examined family relationships and the parenting attitudes of mothers and fathers, respectively, while very few have investigated paternal parenting stress and its consequences for the family and, particularly, for the children (Buss & Plomin, 1984; Kim & Kang, 1997). Although researchers have increasingly considered the role of fathers in recent years (Greenfield et al., 2006), little is still known about the influence of paternal parenting stress on child behavior and the differences between paternal and maternal effects (Bornstein, 2015). The present study focused on both maternal and paternal stress, as it has been shown that fathers are currently more involved in rearing their children than was the case in the past and that mothers and fathers within the same family might be more similar than different in their levels of parenting stress (Ponnet et al., 2013). A recent study (Lee et al., 2018) found that paternal parenting stress had a modest positive association with the development of children’s behavioral problems. On the other hand, McBride et al. (2002) found interesting differences in the role of maternal versus paternal parenting stress on child development, highlighting differences according to child gender. Furthermore, studies have found associations between paternal parenting stress and fathers’ more negative perceptions of their children (Fagan & Lee, 2014; Zelkowitz & Milet, 1997; Miragoli et al., 2018) found both maternal and paternal parenting stress to be related to perceived child behavior. Likewise, research has shown that parenting stress may negatively affect parents’ attitudes toward and interaction with their children (Babore et al., 2019; Fredriksen et al., 2019). While research has pointed out possible differences between the influences of maternal and paternal parental stress on the child’s emotional-behavioral functioning (McBride et al., 2002), several studies have emphasized the importance of considering the influence of parental stress on child development, as factors derived from both parents (Camisasca et al., 2016). As a matter of fact, the research highlighted reciprocal influences of maternal and paternal contributions on child’s development. For example, Malmberg and Flouri (2011) investigated how mothers’ and fathers’ depressed mood and father–child and mother–child relationships predicted preschool children’s problem behavior. Little is known, however, about the mutual influence of maternal and paternal parenting stress on the child’s emotional behavioral development.

In the present study, we considered the mutual contribution of mothers and fathers, and we analyzed clusters based on both mothers’ and fathers’ parenting stress.

## Parenting stress and pre-school children’s emotional behavioral functioning

While most studies have primarily investigated parental psychological distress during the postpartum period, there is a paucity of research examining paternal parenting stress during children’s pre-school age. Chu and Lee (2019) investigated the relationship between parental psychological distress and childcare involvement among fathers of pre-school children, finding an association between higher paternal stress and lower quality and quantity of fathers’ involvement in childcare.

During the pre-school period, children engage in relationships with family members and peers and acquire social skills that help them adapt to their environment (Arslan et al., 2011). As parenting stress may affect children’s ability to learn and experience, it can inhibit their development and skills acquisition, giving rise to behavioral issues (Strauss et al., 2012). In this period, children also increase their language and cognitive skills; the presence of fathers, who tend to be more engaged in play with their children, can be highly beneficial for this development. Indeed, because fathers and mothers tend to have distinct roles in parenting, they are likely to stimulate their children in different ways (Braza et al., 2015).

Given that parenting stress reflects various aspects of parent–child interactions, it can clearly indicate dysfunctions in a family (Ostberg & Hagekull, 2000) and predict behavioral problems in children (Anthony et al., 2005; Mackler et al., 2015; Tafà et al., 2018; Tsotsi et al., 2019). Parents affected by parenting stress may find difficult to manage their children’s needs (Ballarotto et al., 2021; Dubois-Comtois et al., 2013), and they tend to be less efficient in helping their children cope with difficult situations (Nelson et al., 2009; Hustedt et al., 2017) found that parents who reported the highest levels of parenting stress had children who demonstrated more behavioral problems. The family stress model suggests that parents’ perceptions of higher stress may disrupt parent–child relationships, and these disrupted relationships may, in turn, lead to poorer child behavioral outcomes. Consistent with ecological theory (Bronfenbrenner, 1979), there are likely bidirectional influences between family members and their daily spheres of life.

Indeed, it is difficult to determine directionality in the relationship between parental stress and child behavioral outcomes, as most prior studies on this topic have been cross-sectional; however, a few studies have suggested a bidirectional relationship (Barroso et al., 2018; Neece et al., 2012; Woodman et al., 2015). Furthermore, several studies have found associations between parenting stress and children’s behavioral problems (Crum & Moreland, 2017; Gourley et al., 2013; Hastings, 2002); however, most of these studies have relied on parent report-form questionnaires to assess children’s behavioral functioning (Mak et al., 2020; Neece et al., 2012). As highlighted above, parenting stress can impact not only children’s behavior, but also parental perceptions of children’s behavior (Miragoli et al., 2018; Zelkowitz & Milet, 1997). Thus, to manage this potential bias and better understand the role of parenting stress in children’s behavioral functioning, the present study also considered teachers’ views of children’s behavioral functioning. As a matter of fact, numerous studies have highlighted the importance of taking into account the views of multiple informants (Gresham et al., 2018; Miller et al., 2014), especially highlighting the problem of discrepancies between different observers (De Los Reyes & Kazdin, 2005). Specifically relating to parenting stress, Youngstrom et al. (2000) found a positive relation between self-rated parental stress and informant discrepancies on ratings of child internalizing and externalizing problems (Youngstrom et al., 2000).

Previous research has found that positive parent–child relationships and lower parenting stress are associated with fewer informant discrepancies (Cheng et al., 2018; Fung & Lau, 2010; Van der Oord et al., 2006). Moreover, parenting stress has been specifically investigated among the parents of pre-school children affected by ADHD and similar disorders (Baker, 1994; Chen et al., 2017). For example, Rogers et al. (2009) found an association between parenting stress and children with both inattention and hyperactivity/impulsivity symptoms. While some studies have involved clinical samples, few have investigated the impact of parenting stress on children in normative samples. Within samples with clinically diagnosed children, households may have experienced different situations (e.g., different school issues, peer relationship issues, neuropsychological and/or psychological assessments, etc.) that may have uniquely contributed to their levels of parenting stress and/or child behavioral functioning. Thus, research with normative samples may allow for a better understanding of these associations. Accordingly, the present study aimed at investigating the impact of parenting stress on different aspects of children’s behavioral functioning in a non-referred sample.

## The present study

As highlighted above, whereas several studies have reported the effect of maternal parenting stress on children’s functioning, few studies have investigated the role of paternal parenting stress (Pinquart, 2017). As suggested above, fathers and mothers have different roles in parenting and, as a result, they could stimulate their children in different ways (Braza et al., 2015; McBride et al., 2002) found associations between parenting stress and children’s emotional intensity in dyads mother-boy and father-girl. For this reason, the present research aimed to separately investigate the influence of mothers and fathers with different levels of parenting stress on children’s behavioral functioning. Furthermore, in order to better understand the role of parenting stress – linked to parent–child interactions – in shaping parents’ perceptions of their children, the study sought to verify the presence of differences between teachers’ and parents’ views of children’s behavioral functioning. Indeed, the association between parental stress and parent-reported child behavioral functioning could represent the parent’s subjective view influenced by stress related to his or her role. Thus, to minimize the possible bias of parent reports and to better understand how parenting stress might impact children’s behavioral functioning, the present study considered also teachers’ views of children’s behavioral functioning.

Specifically, the research aimed at:

1. testing the presence of differences between maternal and paternal parenting stress on children’s behavioral functioning, as reported by teachers and parents; as suggested by Ponnet et al. (2013), mothers and fathers were hypothesized to have similar levels of parenting stress;
2. verifying the presence of different clusters based on parenting stress, and determining whether parents belonging to different clusters had children with different behavioral functioning; based on several studies (Hustedt et al., 2017; Rogers et al., 2009) parents with higher levels of parenting stress were assumed to have children with more emotional-behavioral difficulties; and.
3. investigating whether parenting stress influenced parents’ perceptions of children’s behavioral functioning; based on Malmberg and Flouri’s study (2011) that investigating mutual influences of maternal and paternal factors on child’s development, we hypothesized parenting stress influencing parental perception of children’s behavioral functioning.

## Methods

### Participants and procedure

Participants were recruited from several Infancy schools in central Italy, where children aged 3–6 years old are all in the same school environment. Upon obtaining permission from the headmasters of the schools for our project, we contacted all the parents of the children, in order to inform them about the research and to ask to consent for them and their child’s teacher to answer questions about the child. Next, the teachers were contacted and detailed information on how to complete the measurements was provided to parents (e.g., parents were instructed to complete the questionnaires independently) and teachers. After the research purposes and procedure were introduced, written informed consent was collected from each participant (parents and teachers) before data collection. All parents and teachers were informed that their participation was voluntary, not compensated and their responses would be anonymous.

Inclusion criteria comprised: (1) parents’ age ≥ 18 years old; (2) parents’ adequate knowledge of the Italian language; (3) parents’ written consent for their own and their child’s teacher participation; and (4) teachers’ knowledge of the child for at least six months. Parents of children with neurological conditions, pervasive developmental disorders and living under local authority care have been excluded.

The parents of *N* = 201 children, aged 3–6 years (51.13% male), with an average age of 4.42 years (*SD* = 0.95), were contacted. All parents accepted their child’s participation in the research but, *N* = 18 mothers and *N* = 25 fathers didn’t return filled-out questionnaires.

Mothers’ average age was 37.17 years (*SD* = 5.36) and fathers’ average age was 40.11 years (*SD* = 5.92). For sociodemographic characteristics, 92% of parents were European and 40.9% of mothers were unemployed. Furthermore, 93.2% of parents were married and 96% of them cohabitated with their partners.

Regarding teachers, *N* = 33 (31 women, 2 men) participated in the study. Teachers’ average age was 48.93 years (*SD* = 7.54) and the average length of experience was 9.95 years (*SD* = 10.6).

During school hours, psychologists and research assistants administered the questionnaires to teachers. Each teacher evaluated between 5 and 7 children. Parents were recruited when they accompanied their children to or picked their children up from school. They were given the questionnaires to complete at home and were asked to return them to the teacher within 2 days. Parents independently completed measures related to parenting stress and both parents and teachers evaluated the problematic behaviors of each child.

The research procedure was fully compliant with APA standards, the Declaration of Helsinki, and the Ethics Code of the Italian Board of Psychology, the regulatory authority that defines national research guidelines.

Data were collected in the second half of the school year, in order to warranty an adequate teachers’ knowledge of the children.

### Measures

The *Parenting Stress Index-Short Form* (PSI-SF; Abidin, 1995; Italian validation from Guarino et al., 2008), a self-report screening questionnaire, was used to assess parental stress due to parental behavior, child characteristics, and/or parent–child interactions. The PSI was designed for use with parents of children aged 1 month to 12 years. The short form of the questionnaire (used in the present study) is composed of 36 items, evaluated on a scale ranging from 1 (*strongly agree*) to 5 (*strongly disagree*).

The PSI-SF investigates three main domains of stressors: *Parental Distress* (PD) measures parents’ level of distress resulting from personal factors directly related to their role (e.g., “I feel trapped by my responsibilities as a parent”); *Parent–Child Dysfunctional Interaction* (P-CDI) measures whether parents perceive their child as not meeting expectations and whether interactions with their children strengthen them as a parent (e.g., “My child rarely does things for me that make me feel very good”); and Difficult Child (DC) measures some fundamental characteristics of child behavior that are more or less easy or difficult to manage and that often originate in the child’s temperament (e.g., “My child makes more demands on me than most children”).

Finally, the total parental stress score, which is calculated by summing all items (possible range: 36–180), indicates the overall level of parental stress. In this study, Cronbach’s alphas for the scales, as administered to mothers and fathers, ranged from 0.79 to 0.87.

The *Strengths and Difficulties Questionnaire* (SDQ; Goodman, 1997) is a report-form questionnaire that is used to investigate parents’ and teachers’ perceptions of children’s behavior. It is used for children aged 4–16 years, but previous studies showed acceptable psychometric properties with 3-year-old children (Cohen & Anders, 2029; Ezpeletaa et al., 2013; Petermann et al., 2010). There is also a version for parents and another self-administered version for adolescents (aged 11–16 years). The SDQ consists of 25 items, classified into five subscales related to children’s behavior: *Emotional Symptoms* (e.g., “Many worries, often seems worried”), *Conduct Problems* (e.g., “Often has temper tantrums or hot tempers”), *Hyperactivity/Inattention* (e.g., “Restless, overactive, cannot stay still for long”), *Peer Relationship Problems* (e.g., “Rather solitary, tends to play alone”), and *Prosocial Behavior* (e.g., “Shares readily with other children – treats, toys, pencils, etc.”).

Answers are reported on a 3-point Likert scale ranging from 1 (*not true*) to 3 (*certainly true*). Respondents are asked to mark the answer that best describes the child’s behavior in the last 6 months. In this study, Cronbach’s alphas for the scales, as administered to teachers, mothers, and fathers, ranged from 0.46 to 0.79. The only scale that showed a value below the reliability threshold (alpha < 0.70) was the *Peer Relationship Problems*, in both fathers’ and mothers’ questionnaires (0.46 and 0.52 respectively). This scale had also shown low reliability in the Italian validation of the instrument for parents (Tobia & Marzocchi, 2018).

The *Social Competence and Behavior Evaluation Scale* (SCBE-30; LaFreniere & Dumas, 1996), is a 30-item questionnaire that evaluates teachers’ views on the quality of a child’s relationships with teachers and peers. In particular, it records children’s social skills, negative behaviors, and emotional problems. The scale (which derives from the original SCBE-80 version) is composed of three subscales: two concerning maladaptive behavior (*Anger/Aggression* and *Anxiety/Withdrawal*) and one referring to adaptive behavior (*Social Competence*). Each subscale includes 10 items.

The Anger/Aggression subscale reflects children’s aggression, anger, and opposing behavior (e.g., “Irritable, gets mad easily”; “Defiant when reprimanded”). Children with a high score in this subscale demonstrate negative feelings and an inability to regulate negative emotions. The Anxiety/Withdrawal subscale assesses shyness, anxiety, and isolation behaviors (e.g., “Remains apart, isolated from the group”; “Doesn’t talk or interact during group activities”). Children with a high score in this subscale spend a lot of time alone and show anxiety when they are involved in group activities. Finally, the Social Competence subscale measures positive social interactions and prosocial behavior with peers (e.g., “Works easily in groups”; “Cooperates with other children”). Children with good social behavior show positive feelings in their interactions and are appreciated by peers and teachers.

Teachers are asked to indicate the frequency with which the indicated behaviors are “usually” present in the child, according to a 6-point Likert scale ranging from 1 (*never*) to 6 (*always*). In this study, Cronbach’s alphas ranged from 0.69 to 0.78.

### Data analysis

A preliminary screening of the data showed few data missing for each instrument (2% for each instrument). Missing data were corrected using multiple imputations in SPSS software (Version 27.0).

Descriptive statistics were performed (i.e., reliabilities, frequencies, mean scores, percentages). Furthermore, to verify differences between mothers and fathers on parenting stress, a univariate analysis of variance (ANOVA) was conducted. A second ANOVA was run to verify possible differences between parents and teachers on their views of children’s behavioral functioning.

A cluster analysis was carried out to verify the existence of specific clusters based on mothers’ and fathers’ parenting stress. Adopting the methodology of Arabie and Hubert (1992), groups of participants were formed with minimal within-group variability (i.e., group members were as similar as possible) and maximal between-group variability (i.e., group members were as different as possible from those in other groups). First, an agglomerative hierarchical cluster analysis was conducted to establish the number (k) of clusters in the sample. Then, a k-means (non-hierarchical) cluster analysis procedure was used to find the optimal k class solution (with k deriving from the hierarchical step). Following this, an ANOVA was used to verify whether parents belonging to different clusters (according to the level of parenting stress) had children with different behavioral functioning.

Finally, after verifying the association between variables, a multiple regression analysis was run to investigate whether parenting stress is associated with mothers’ and fathers’ perceptions of children’s behavioral functioning. Specifically, the dependent variables were parents’ SDQ scores, and the independent variables were mothers’ and fathers’ PSI-SF scores.

All data were analyzed using IBM SPSS statistics version 25.

## Results

### Maternal and paternal parenting stress

To verify the presence of differences between maternal and paternal parenting stress, a univariate analysis of variance (ANOVA) was conducted on all PSI-SF scores. The results showed that mothers scored higher on parenting distress, *F*(1,346) = 5.55, *p* = .019, partial η2 = 0.02, and total stress, *F*(1,346) = 3.66, *p* = .056, partial η2 = 0.01, relative to fathers. Table 1 displays all the ANOVA results.

**Table 1:** Average scores, standard deviations and F of mothers’ and fathers’ parenting stress

	Mothers	Fathers	*F* (1,346)	*p*
PD	23.19 (7.58)	21.37 (6.8)	5.55	0.019
P-CDI	18.01 (5.58)	17.3 (4.52)	1.72	0.191
DC	23.05 (7.15)	22.27 (6.77)	1.07	0.302
Total Parenting Stress	64.25 (17.1)	60.94 (15.06)	3.66	0.056

Note. PD = Parental Distress; P-CDI = Parent-child Dysfunctional Interactions; DC = Difficult Child

### Children’s behavioral functioning, as reported by teachers and parents

An ANOVA was carried out to detect differences between mothers’, fathers’, and teachers’ perceptions of children’s behavioral functioning. The results showed a significant difference in prosocial behavior, *F*(2,523) = 8.8, p = .000, partial η2 = 0.03, whereby mothers and fathers reported more prosocial behavior in children, relative to teachers, *p* < .001 (see Table 2).

**Table 2:** Average scores, standard deviations and F of mothers’, fathers’ and teachers’ perceptions of children’s behavioral functioning

	Mothers	Fathers	Teachers	*F* (2,523)	*p*
Emotional symptoms	1.57 (1.45) ^a^	1.21 (1.36) ^a^	1.53 (1.65) ^a^	3.11	0.072
Conduct problems	1.72 (1.54) ^a^	1.39 (1.48) ^a^	1.4 (1.82) ^a^	2.35	0.097
Hyperactivity	2.44 (1.82) ^a^	2.54 (1.86) ^a^	2.74 (2.43) ^a^	0.976	0.378
Peer relationships	1.34 (1.46) ^a^	1.28 (1.48) ^a^	1.31 (1.61) ^a^	0.067	0.936
Prosocial behavior	7.86 (1.68) ^a^	7.76 (1.73) ^a^	7.05 (2.41) ^b^	8.8	0.000
Total Difficulties	7.06 (4.45) ^a^	6.41 (4.6) ^a^	6.98 (5.4) ^a^	0.935	0.393

### Cluster analysis based on parenting stress

Mothers and fathers with different levels of parenting stress, grouped in specific clusters, were associated with children with different behavioral functioning.

A k-means cluster analysis was performed to classify participants into groups on the basis of maternal and paternal parenting stress. An agglomerative hierarchical cluster analysis was then conducted to determine the number of clusters. Ward’s (1963) method with squared Euclidean distances was used to test solutions for two-clusters through seven-clusters, as suggested by Milligan and Sokol (1980). Based on the scree plot, the most interpretable pattern resulted in a three-cluster solution, which maximized both the homogeneity of individuals within clusters and the heterogeneity of individuals between clusters. Based on this three-cluster solution, a k-means cluster analysis was computed.

The final cluster solution identified three distinct profiles based on maternal and paternal parenting stress. Table 3 shows the average scores for each cluster, highlighting distinct differences between them.

**Table 3:** Average scores and differences between maternal and paternal PSI-SF scales based on the three Clusters identified

	Cluster 1 (*High stressed parents*; 13,37% of the sample)	Cluster 2 (*Middle stressed parents*; 38,37% of the sample)	Cluster 3 (*Low stressed parents*; 48,26% of the sample)	*F* (169,2)	*p*
Maternal PD	34.09 (6.55) ^a^	26.08 (4.98) ^b^	17.77 (4.13) ^c^	122.09	0.000
Maternal P-CDI	27.35 (6.73) ^a^	18.48 (3.75) ^b^	14.81 (2.53) ^c^	99.51	0.000
Maternal DC	32.61 (6.76) ^a^	24.8 (5.04) ^b^	18.75 (5.16) ^c^	66.91	0.000
Paternal PD	24.91 (7.88) ^a^	25.74 (4.95) ^a^	16.92 (4.63) ^b^	57.29	0.000
Paternal P-CDI	20.96 (6.17) ^a^	18.76 (4.24) ^a^	15.12 (2.8) ^b^	26.85	0.000
Paternal DC	27.83 (9.05) ^a^	25.11 (4.89) ^a^	18.48 (4.99) ^b^	38.08	0.000

Note. PD = Parental Distress; P-CDI = Parent-child Dysfunctional Interaction; DC = Difficult Child

Means in rows, not sharing a common letter, differ significantly (p < .05).

As is evident, the three clusters were composed of different levels of maternal and paternal parenting stress. Cluster 1 (“high stressed parents”) was described by higher levels of maternal parenting stress and middle levels of paternal parenting stress; cluster 2 (“middle stressed parents”) was characterized by middle levels of both maternal and paternal stress; and cluster 3 (“low stressed parents”) demonstrated low levels of maternal and paternal parenting stress.

An ANOVA was conducted to verify the presence of differences in children’s behavioral functioning with respect to parents in different clusters. For this purpose, teachers’ reports of children’s behavioral functioning were considered, through the SDQ and SCBE-30 questionnaires. The results showed significant differences in children’s prosocial behavior, *F*(2,169) = 3.71, *p* = .026, partial η2 = 0.04, whereby middle stressed parents (i.e., cluster 2) had children with more prosocial behavior than did other parents, *p* < .05. It is interesting to note that this difference was not observed between high and low stressed parents.

As regards the SCBE-30 scales, no differences emerged between clusters.

### Associations between parenting stress and children’s behavioral functioning, as reported by different informants

The presence of associations between maternal and paternal parenting stress and children’s behavioral functioning, as reported by parents and teachers, was verified. Specifically, Table 4 shows the correlation coefficients between mothers’ and fathers’ PSI-SF scales and mothers’, fathers’, and teachers’ SDQ scales.

**Table 4:** Pearson coefficients of correlations between maternal and paternal parenting stress and parents’ and teachers’ views of children’s behavioral functioning

	M_PD	M_P-CDI	M_DC	M_DEF	F_PD	F_P-CDI	F_DC	F_DEF
M_Emot	0.20**	0.29**	0.34**	0.22**	0.01	− 0.02	0.18*	0.13
M_Cond	0.22**	0.21**	0.44**	0.26**	0.29**	0.34*	0.47**	0.31**
M_Hyper	0.25**	0.29**	0.43**	0.25**	0.19*	0.27**	0.35**	0.21**
M_Peer	0.13	0.25**	0.23**	0.13	0.04	0.12	0.19*	0.08
M_Prosoc	− 0.30**	− 0.23**	− 0.22**	− 0.30**	− 0.22*	− 0.27**	− 0.20**	− 0.20**
F_Emot	0.13	0.25**	0.36**	0.09	0.31**	0.26**	0.39**	0.31**
F_Cond	0.18*	0.26**	0.32**	0.16*	0.19*	0.27**	0.42**	0.21**
F_Hyper	0.19*	0.31**	0.41**	0.17*	0.20**	0.30**	0.43**	0.21**
F_Peer	0.17*	0.26**	0.28**	0.17*	0.25**	0.19*	0.21**	0.29**
F_Prosoc	− 0.22**	− 0.30**	− 0.17*	− 0.20**	-0.06	− 0.17*	− 0.18*	− 0.07
T_Emot	0.04	0.08	0.14	0.11	0.05	0.05	0.11	0.06
T_Cond	0.04	0.08	0.13	0.14	0.07	0.07	0.05	0.11
T_Hyper	0.03	0.06	0.15*	0.07	0.06	0.09	0.13	0.10
T_Peer	0.06	0.09	0.15*	0.06	0.01	0.03	0.00	− 0.01
T_Prosoc	-0.06	-0.04	-0.08	− 0.05	-0.02	-0.05	-0.07	− 0.06

Note. M = Mother; F = Father; T = Teacher; PD = *Parental Distress*; P-CDI = *Parent-child Dysfunctional Interactions*; DC = *Difficult Child*; Emot = *Emotional symptoms subscale;* Cond = *Conduct problems subscale;* Hyper = *Hyperactivity/inattention subscale;* Peer = *Peer relationships problem subscale;* Prosoc = *Prosocial behavior subscale*

* *p* < .05

** *p* < .01

As is evident, parenting stress correlated with parents’ own views of their children’s behavioral functioning, but not those of teachers. Based on these results, multiple regression analyses were conducted to verify whether parenting stress affected subsequent perceptions of children’s behavioral functioning. In particular, the dependent variables were parents’ SDQ scores, and the independent variables were mothers’ and fathers’ PSI-SF scores. Specifically, the dependent variables were parents’ SDQ scores, and the independent variables were mothers’ and fathers’ PSI-SF scores. Regression analyses were conducted for each maternal and paternal SDQ subscale (dependent variables). In each regression analyses, the three PSI-SF subscales were included as independent variables. Child’s sex was included as a covariate.

First, multiple regression analyses were conducted to verify whether maternal parenting stress influenced mothers’ perceptions of their children’s behavioral functioning. The results showed that the DC score predicted the following SDQ scores, as compiled by mothers: Emotional Symptoms, *R*^*2*^ = 0.125, β = 0.25, *T* = 2.68, *p* < .01, Conduct Problems, *R*^*2*^ = 0.197, β = 0.49, *T* = 5.41, *p* < .001, and Hyperactivity/Inattention, *R*^*2*^ = 0.19, β = 0.412, *T* = 4.49, *p* < .001. Furthermore, the DC score also influenced children’s total problems score, *R*^*2*^ = 0.27, β = 0.47, *T* = 5.37, *p* < .001.

To verify whether maternal parenting stress influenced fathers’ perceptions of children’s behavioral functioning, multiple regression analyses were conducted. The results showed that mothers’ DC scores predicted fathers’ scores on the following SDQ scales: Emotional Symptoms, *R*^*2*^ = 0.14, β = 0.36, *T* = 3.83, *p* < .001, Conduct Problems, *R*^*2*^ = 0.11, β = 0.26, *T* = 2.73, *p* < .01, Hyperactivity/Inattention, *R*^*2*^ = 0.18, β = 0.38, *T* = 4.17, *p* < .001, and Peer Relationship Problems, *R*^*2*^ = 0.09, β = 0.20, *T* = 2.10, *p* < .05. On the other hand, the results also showed that mothers’ scores on the P-CDI scale predicted fathers’ scores on the SDQ Prosocial Behavior subscale, *R*^*2*^ = 0.09, β = − 0.28, *T* = -2.87, *p* < .01.

As regards paternal parenting stress, a multiple regression analysis was conducted to verify whether it predicted fathers’ perceptions of children’s behavioral functioning. The results showed that paternal DC predicted the following SDQ scores, as compiled by fathers: Emotional Symptoms, *R*^*2*^ = 0.17, β = 0.33, *T* = 3.49, *p* < .01, Conduct Problems, *R*^*2*^ = 0.18, β = 0.43, *T* = 4.56, *p* < .001, and Hyperactivity/Inattention, *R*^*2*^ = 0.18; β = 0.40, *T* = 4.26, *p* < .001. Furthermore, the DC score influenced children’s total problems score, *R*^*2*^ = 0.26, β = 0.43, *T* = 4.84, *p* < .001, and fathers’ PD score influenced their Peer Relationship Problems score, *R*^*2*^ = 0.07, Beta = 0.19, *T* = 2.11, *p* < .05.

Finally, multiple regression analyses were conducted to verify whether paternal parenting stress influenced mothers’ perceptions of children’s behavioral functioning. The results showed that fathers’ DC scores predicted mothers’ scores on the following SDQ scales: Emotional Symptoms, *R*^*2*^ = 0.07, β = 0.31, *T* = 3.07, *p* < .05, Conduct Problems, *R*^*2*^ = 0.23, β = 0.41, *T* = 4.52, *p* < .001, Hyperactivity/Inattention, *R*^*2*^ = 0.13, β = 0.29, *T* = 3.02, *p* < .01, and Peer Relationship Problems, *R*^*2*^ = 0.04, β = 0.22, *T* = 2.19, *p* < .05. Moreover, the results showed that paternal parenting stress, as determined by the P-CDI subscale, predicted mothers’ scores on the Emotional Symptoms subscale, *R*^*2*^ = 0.07, β = − 0.25, *T* = -2.5, *p* < .05, and the Prosocial Behavior subscale, *R*^*2*^ = 0.08, β = − 0.21, *T* = -2.09, *p* < .05.

## Discussion

Several studies have only reported the role of maternal parenting stress or the joint effect of maternal and paternal parenting stress (Phares et al., 2005) on children’s development. The present study aimed to separately investigate the association between mothers and fathers with different levels of parenting stress (grouped into specific clusters) and children with different behavioral problems.

The results suggest that parenting stress may affect children’s ability to learn and experience and therefore inhibit their development and skills acquisition, giving rise to behavioral issues (Abidin, 1992). Furthermore, as evidenced by previous studies (Cheng et al., 2018), the research focused on the association between measures of parenting stress and parents’ and teachers’ perceptions of children’s problems.

Specifically, the first objective was to verify the presence of differences between maternal and paternal parenting stress. In contrast to our hypothesis, the results showed that mothers had higher levels of parenting stress than did fathers. Interestingly, although mothers and fathers did not differ in their perceptions of parenting stress relative to their interactions with children (P-CDI) or their children’s behavior (DC), mothers perceived greater parenting distress (PD). The results show that mothers and fathers have similar levels of stress in interacting with their children, but mothers experience higher levels of stress in assuming their parental role. This could also depend on the division of roles within parenting pairs. In fact, Ponnet et al. (2013) pointed out that currently, fathers experience similar levels of parenting stress to mothers in that there is greater equalization of parenting roles than in the past. It would be important to be able to investigate more how much the division of roles within the family may be related to perceived parental stress. In order to better understand this data, further analysis was carried out to assess the relationship between study variables (i.e., the third study objective).

The study also sought to verify the presence of differences between teachers’ and parents’ views of children’s behavioral functioning. Unlike our assumptions, results found no differences emerged between the parent and teacher reports, except with respect to children’s prosocial behavior, which parents tended to rate higher than teachers. This result is very interesting: although parents and teachers reported similar views of children’s emotional and behavioral difficulties, parents saw more prosocial behavior in their children. This may be due to the different observation contexts of home and school. The school environment provides a broad context for socialization, where children learn social values in interaction with peers and adults. In particular, Lemos and de Richaud (2014) and Flouri and Sarmadi (2016) have underlined the role played by the social context in shaping children’s prosocial behavior. On the other hand, in the school context, the many interactions in which children are immersed may make it more difficult for pro-social behaviors to emerge and/or make it more difficult for teachers to catch these behaviors. In line with this, more opportunities for one-to-one interaction in the family may facilitate prosocial behavior or the internalization of the prosocial behaviors depending on the quality of the parent-child interaction may be more possible.

The second research objective was to verify whether parents with different levels of parenting stress had children with different behavioral functioning. A k-means cluster analysis was carried out on maternal and paternal parenting stress, and three clusters were identified: high, middle and low stressed parents. Differences in children’s behavioral functioning was verified between clusters, whereby middle stressed parents had children with more prosocial behavior than did parents in other clusters. It is interesting to note that no differences were found between children in the high versus the low stressed parent clusters. These results underline that parents with average levels of parenting stress had children with more prosocial behavior than did both stressed parents and parents reporting lower levels of parenting stress. It seems that the parents that are able to better regulate parenting stress are the parents of children with more prosocial behavior – and therefore greater openness to others. In line with Shaver et al.’s (2010) behavioral-systems perspective, we consider prosocial behavior to be rooted in the caregiving system, whereby the quality of the parent–child relationship may be associated with children’s prosocial behavior, empathy, and compassion, which represent important protective factors in their psycho-emotional development.

Finally, the third study objective was to investigate whether parenting stress was linked to perceptions of children’s behavioral functioning. First, the possible presence of associations between maternal and paternal parenting stress and children’s behavioral functioning, as reported by parents and teachers, was investigated. A correlation analysis was carried out and the results (see Table 4) indicated that parents’ perceptions of children’s behavioral functioning were associated with their level of parenting stress. On the other hand, the latter was not associated with teachers’ perceptions of children’s behavioral functioning.

It is very interesting that parenting stress was associated with parents’ perceptions but not with teachers’ ones, as this suggests that parents’ perceptions of their children may be linked to their difficulties interacting with them. Furthermore, no significant differences were found between the scores reported by mothers, fathers, and teachers, except on the Prosocial Behavior scale. This may be explained by the fact that the study considered a normative sample; further studies involving clinical samples are needed to clarify this difference. Despite this, to better understand these dynamics, multiple regression analyses were conducted to investigate whether parenting stress influenced mothers’ and fathers’ perceptions of children’s behavioral functioning. The results showed that parenting stress was associated with different aspects of children’s behavioral functioning. Specifically, it is interesting to observe some similarities between maternal and paternal parenting stress and child behavioral functioning. In particular, both maternal and paternal parenting stress with regard to the perception of the child as difficult are associated with greater emotional and behavioral problems in the children. However, the regression coefficients were not very strong, indicating that parenting stress was only a partial predictor of children’s behaviors. Indeed, although Anthony et al. (2005) found that higher parenting stress increased children’s externalizing behavioral problems, Beck et al. (2004) found that children’s behavioral problems predicted parenting stress. Thus, the literature on this matter is mixed, and Neece et al. (2012) suggested the existence of a bidirectional relationship between mothers’ and fathers’ parenting stress and children’s behavior problems. Furthermore, in line with the current literature (Kochanova et al., 2022; Rodriguez et al., 2019; Tokunaga et al., 2019), the present results also showed that parenting stress seems to be linked to partners’ perceptions of children’s behavioral functioning. However, as mentioned above, despite rigorous studies, the literature is inconsistent regarding of unidirectional or bidirectional associations between parenting stress and children’s behavior problems (Jiang et al., 2022).

Further research is necessary to better understand the complex dynamics highlighted in this study also in light of the consequences of the current Covid-19 pandemic on family and school contexts as shown by recent studies (Babore et al., 2021; Scarpellini et al., 2021).

More specifically, a deeper investigation of the role of children’s temperamental variables is needed to understand the relationships between the investigated variables and of the role of the couple’s dyadic adjustment (Velotti et al., 2011). This speaks to one limitation of the present study. A second limitation involves the use of self-report and report-form questionnaires, which did not facilitate a completely objective evaluation of children. Furthermore, it is important to highlight that groups of children were assessed by different teachers (N = 33). This difference could create a bias in the observations. On the other hand, the possibility of having evaluations from different teachers makes it possible to reduce possible subjective influences in the evaluation.

On the other hand, a strength of the study involved the multiplicity of child observers, which helped to uncover how parenting stress, linked to parent–child interaction, may play an important role in shaping parents’ perceptions of their children. Indeed, this result may encourage future research to employ more appropriate tools to measure children’s emotional and behavioral characteristics, ensuring that such assessments are less influenced by parenting stress (as in the case of parent reports). A further strength of this study concerned its investigation of parenting stress and children’s behavioral functioning in a non-risk situation, enabling a better understanding of the variations that exist in parent–child dynamics.

This study showed an association between parenting stress and partners’ perceptions of children’s behavioral functioning. The results of the current study may have practical implications for research and clinical practice. In fact, very often research assessed the child’s emotional-behavioral functioning through report-form questionnaires filled out by parents, often with evaluation bias. In order to have a more objective assessment of the child’s behavioral functioning, it is suggested to have information from several observers of the child (e.g., parents, teachers, educators etc.), or to have an assessment from a specifically trained observer.

## Data Availability

The data are available on request by email to the correspondent author.

## Data accessibility statement

The data presented in this study are openly available in FigShare at doi: 10.6084/m9.figshare.16885264.
